# The endogenous transposable element *Tgm9* is suitable for generating knockout mutants for functional analyses of soybean genes and genetic improvement in soybean

**DOI:** 10.1371/journal.pone.0180732

**Published:** 2017-08-10

**Authors:** Devinder Sandhu, Jayadri Ghosh, Callie Johnson, Jordan Baumbach, Eric Baumert, Tyler Cina, David Grant, Reid G. Palmer, Madan K. Bhattacharyya

**Affiliations:** 1 USDA-ARS, US Salinity Laboratory, Riverside, CA, United States of America; 2 Department of Agronomy, Iowa State University, Ames, IA, United States of America; 3 Department of Biology, University of Wisconsin-Stevens Point, Stevens Point, WI, United States of America; 4 USDA-ARS Corn Insects and Crop Genomics Research Unit, Ames, IA, United States of America; Ben-Gurion University, ISRAEL

## Abstract

In soybean, variegated flowers can be caused by somatic excision of the CACTA-type transposable element *Tgm9* from Intron 2 of the *DFR2* gene encoding dihydroflavonol-4-reductase of the anthocyanin pigment biosynthetic pathway. *DFR2* was mapped to the *W4* locus, where the allele containing *Tgm9* was termed *w4-m*. In this study we have demonstrated that previously identified morphological mutants (three chlorophyll deficient mutants, one male sterile-female fertile mutant, and three partial female sterile mutants) were caused by insertion of *Tgm9* following its excision from *DFR2*. Analyses of *Tgm9* insertion sites among 105 independent mutants demonstrated that *Tgm9* hops to all 20 soybean chromosomes from its original location on Chromosome 17. Some genomic regions are prone to increased *Tgm9*-insertions. *Tgm9* transposed over 25% of the time into exon or intron sequences. *Tgm9* is therefore suitable for generating an indexed insertional mutant collection for functional analyses of most soybean genes. Furthermore, desirable *Tgm9*-induced stable knockout mutants can be utilized in generating improved traits for commercial soybean cultivars.

## 1. Introduction

The soybean genome has been sequenced and expression patterns of most soybean genes are known [1, 2]. Although putative genes have been predicted based on the DNA sequence and when possible annotated for inferred function based on protein homology, many soybean genes remain uncharacterized even at this level of annotation. Rapid identification of biological functions of soybean genes will require an indexed insertional mutant collection suitable for reverse genetics studies.

Gene silencing through RNAi has been successfully used in the functional characterization of plant genes [3]. However, the incomplete inactivation of target genes is a common problem with such gene silencing approaches and makes the data interpretation difficult [4]. T-DNA insertion mutagenesis, in which insertion of T-DNA into the coding or promoter sequence of a gene can disrupt its function, has been effectively utilized in Arabidopsis [5–7]. Gene editing has been shown to be a powerful approach for the functional analyses of genes in plant species including maize [8, 9]. In soybean, the main bottleneck associated with the application of RNAi, T-DNA insertion mutagenesis or gene editing approaches for functional analyses of a large number of genes is the lack of a high-throughput transformation procedure and availability of large greenhouse spaces for growing transformants.

Transposable elements are a major component of the genomes of higher eukaryotes and are widely distributed among plant species [10]. Functional characterization of thousands of soybean genes could be facilitated by knockout mutants induced by transposons [11–14]. Active endogenous transposable elements have been identified in several plant species and have been effectively used in functional characterization of plant genes [15, 16].

The utility of active transposons from maize (*Ac/Ds*), rice (*mPing*) and tobacco (*Tnt1*) in tagging soybean genes has been demonstrated [11–13]. However, this approach requires genetic transformation. Handling thousands of transgenic lines in the field prior to deregulation is impractical and cumbersome. Furthermore, products generated through genetic transformation are often not well received by a significant proportion of the end users worldwide and the deregulation step could be lengthy and expensive. Tagging of soybean genes using an endogenous transposable element is therefore an attractive solution not only for functional analyses of tens of thousands of soybean genes, but also in generating desirable mutants for rapid genetic improvement of important traits in the commercial soybean cultivars. Since mutants created by an endogenous transposon are not GMOs, the products produced from incorporation of such mutants should be accepted by all consumers.

Five genes, *W1*, *W3*, *W4*, *Wm*, and *Wp*, regulate pigmentation in flowers and hypocotyls of soybean [17]. The mutant *w4-m* allele at the *W4* locus is characterized by altered pigment accumulation patterns in flowers and hypocotyls [18]. The soybean line with *w4-m* allele was added to soybean collection and assigned the genetic type collection number T322 [19]. *W4* contains the *DFR2* gene encoding a functional dihydroflavonol-4-reductase 2 enzyme. Somatic excision of a 20,548-bp CACTA-type transposable element, *Tgm9*, from Intron 2 of *DFR2* causes variegated flowers and hypocotyls with dark brown sectors [14]. Since *Tgm9* is located in an intron, both precise and imprecise excisions of the element lead to restoration of the wild-type phenotype. Excision of the element from the germ tissues of the parent plant results in at least some progenies that carry only purple flowers and are termed germinal revertants. The mutation rates in several genetic loci among the revertants with purple flowers were much higher than the rate of spontaneous mutation [20]. Therefore, it was hypothesized that following excision of *Tgm9* from *DFR2*, the element inserts into new genetic loci.

*Tgm9* excises at a high rate with a germinal reversion frequency of about 6% per generation [21]. Several mutant genes, unlinked to the *W4* locus, were isolated by screening germinal revertants for morphological mutant phenotypes [20, 22, 23]. For example, male-sterility, female-sterility and root necrotic root mutants were identified by screening thousands of germinal revertants carrying purple flowers [24, 25]. The present study was undertaken to determine if *Tgm9* is suitable for creating knockout mutants for conducting large-scale functional analyses in soybean.

## 2. Materials and methods

### 2.1. Genetic material

Seeds of chlorophyll deficient mutants (Genetic type collection numbers T323, T325 and T346), male-sterile, female-fertile mutant (T359) and partial female sterile mutants (T364, T365 and T367) were obtained from Dr. Randall Nelson, USDA-Agricultural Research Services.

For generating germinal revertants for this study, we identified T322 (*w4-m*) plants that showed variegated flowers and their progenies were grown to identify germinal revertants. One germinal revertant per progeny row was selected for this study (S1 Fig). Leaf tissues from each of the selected germinal revertants was harvested for DNA preparation according to a previously described method [26].

### 2.2. Transposon display

A GenomeWalker Universal kit (Clontech Laboratories, Inc., Mountain View, CA, USA) was used to find the unknown genomic DNA sequences adjacent to a transposable element using the manufacturer’s instructions. DNA (2.5 μg) was independently digested with four restriction enzymes (*Dra*I, *Eco*RV, *Pvu*II, and *Stu*I) to generate blunt ended fragments (S2 Fig). After phenol:chloroform::1:1 purification, digested genomic DNA was ligated to the GenomeWalker adaptor to generate genomic DNA libraries. The four genomic libraries, generated through digestion of DNA with four restriction endonucleases for each germinal revertant were used for the first PCR (PCR 1) using the outer adaptor primer (AP1) and an outer *Tgm9*-specific primer (Trans R1) (S2 Fig; S1 Table). The PCR 1 mixture was then diluted to 100 times and used as a template for a second or “nested” PCR (PCR 2). PCR 2 reactions were conducted using the nested adaptor primer (AP2) and a nested *Tgm9*-specific primer (Trans R2) (S2 Fig). For visualization, the resulting PCR products were separated on a 1.5% agarose gel at 100 V for 1 hour. As there are some residual/truncated copies of *Tgm9* in T322, only unique bands of individual mutants were sequenced (S2 Fig). Residual/truncated copies were displayed as common bands among the selected mutants (S3 Fig). Unique PCR products were sequenced and evaluated for presence of over 100 bp *Tgm9*-end specific sequences.

### 2.3. Sequencing of the transposon inserted into the soybean male-sterile, female-sterile *GmMER3* gene

Long range PCR was performed to amplify a region spanning 2,523 base pairs of the 5’-end of the transposon in two steps using two nested primers from *Tgm9* and two from the mutant *MER3* gene of *mer3* (S1 Table). The PCR products were amplified using Phusion high fidelity DNA polymerase (Thermo Fisher, USA). Bands of correct size were extracted from a 0.8% agarose gel using IBI gel extraction kit (IBI Scientific, USA). The samples were sequenced using the inner *Tgm9* and inner *MER3* primers at the DNA Facility at Iowa State University. The resulting sequence was aligned with the *Tgm9* (GQ344503.1) and Tgmt* (EU190440.1) sequences.

### 2.4. Identification of *Tgm9*-insertion sites in the soybean genome

*Tgm9*-insertion mutants were named using an initial 'T9' with a nomenclature similar to that used for other mutant types in SoyBase. The location of each *Tgm9* insertion was identified by using BLAST to find the location of the element's flanking sequence. The location was determined in both the Wm82.a1 and Wm82.a2 soybean genome assemblies (http://soybase.org/aboutgenomenomenclature.php). The proportion of exons and introns in the soybean genome sequences that were mapped to chromosomes were calculated by considering the predicted exonic and intronic sequences in the Wm82.a2 genome assembly (1).

### 2.5. Calculation of exon and intron sequences of the soybean genome

The data in the GFF file used at SoyBase for the Wm82.a2 genome assembly was parsed into the component parts of the genes. For each gene we used the longest splice variant and determined (i) the full length of the gene, i.e. the region transcribed; (ii) the length of the CDS, i.e. the translated mRNA from ATG to TAG with introns removed; (iii) the length of the 5’ UTR (un-translated region) in the translated mRNA, i.e. with any introns removed; (iv) the length of the 3’ UTR in the translated mRNA, i.e. with any introns removed; (v) the combined length of all introns, i.e. those sequences spliced out of the 5’ UTR and/or the translated region and/or the 3’ UTR. These values were summed for all gene models in the Wm82.a2 genome assembly. We used the sum of the 5’ UTR, CDS and 3’ UTR lengths as the total exon length in the genome. The proportions of exon (11.6%) and intron (10.5%) sequences (S2 Table) are used as the expected values of random *Tgm9* insertion into the soybean genome.

### 2.6. Statistical analysis

We conducted the χ^2^ analysis to determine if the observed insertion of *Tgm9* into exons and introns was random. χ^2^ values were calculated for both classes of insertions; exon and intron-specific insertions with a degree of freedom of 1.

### 2.7. Deposition of data and availability of supporting data

Most of the data are presented in the manuscript or as supplemental data files. The data for the *Tgm9* insertion sites have been deposited to SoyBase and are available at http://soybase.org/gb2/gbrowse/gmax2.0x/?start=1;stop=56831624;ref=Gm01;width=1024;version=100;flip=0;grid=1;id=28f86f22137273c155fe3e1756259be0;l=tgm9-gene_models_wm82_a2_v1-pericentromere%3Aoverview.

## 3. Results

We investigated the applicability of *Tgm9* in generating knockout mutants for functional analyses of soybean genes as follows. First, we determined if the insertion following excision of *Tgm9* from the mutable *w4-m* allele resulted in mutation among the mutants previously identified from screening germinal revertants, generated from the T322 line [20, 23, 27–31] (Table 1). Second, we investigated 124 random germinal revertants to determine the properties of *Tgm9* transposition.

**Table 1:** Genetically mapped soybean mutants identified by screening germinal revertants of the mutable line, T322 (*w4-m*).

Name of line	Mutant name	Mutant phenotype	Reference	Chromosome location	Associated markers	Reference
T323	*y20 (Ames 2) mdh1-n (Ames 2)*	Yellow-green leaves, malate dehydrogenase 1 null	[27]	12	Satt 253 and Satt 279	[29]
T325	*y20 (Ames 4) mdh 1-n (Ames 4)*	Yellow-green leaves, malate dehydrogenase 1 null	[27]	12	Satt 253 and Satt 279	[29]
T346	*y20 (Ames 17) mdh1-n (Ames 19)*	Yellow-green leaves, malate dehydrogenase 1 null	[23]	12	Satt 253 and Satt 279	[29]
T359	*ms9*	Male sterile-female fertile	[28]	3	Satt 521 and Satt 237	[30]
T364	*fsp2*	Female partial sterile	[20]	6	Satt 170 and Satt 363	[31]
T365	*fsp3*	Female partial sterile	[20]	8	Satt429 and Satt 538	[31]
T367	*fsp5*	Female partial sterile	[20]	18	Satt 324 and Satt 138	[31]

### 3.1. *Tgm9* induces mutations following excision from *DFR2*

Several morphological mutants were previously identified from screening thousands of germinal revertants generated from the T322 (*w4-m*) mutable line. These mutants include chlorophyll deficient mutants (T323, T325 and T346) [23, 27], male-sterile, female-fertile mutant (T359) [28] and partial female sterile (*fsp*) mutants (T364, T365 and T367) [20] (Table 1). The genes responsible for the mutant phenotypes in these mutants have been previously genetically mapped [29–31]. To determine if the mutations in these mutants were caused by *Tgm9*-insertion, we identified the *Tgm9*-insertion sites using a transposon display approach for each of the mutants (S2 Fig).

**Fig 1:**
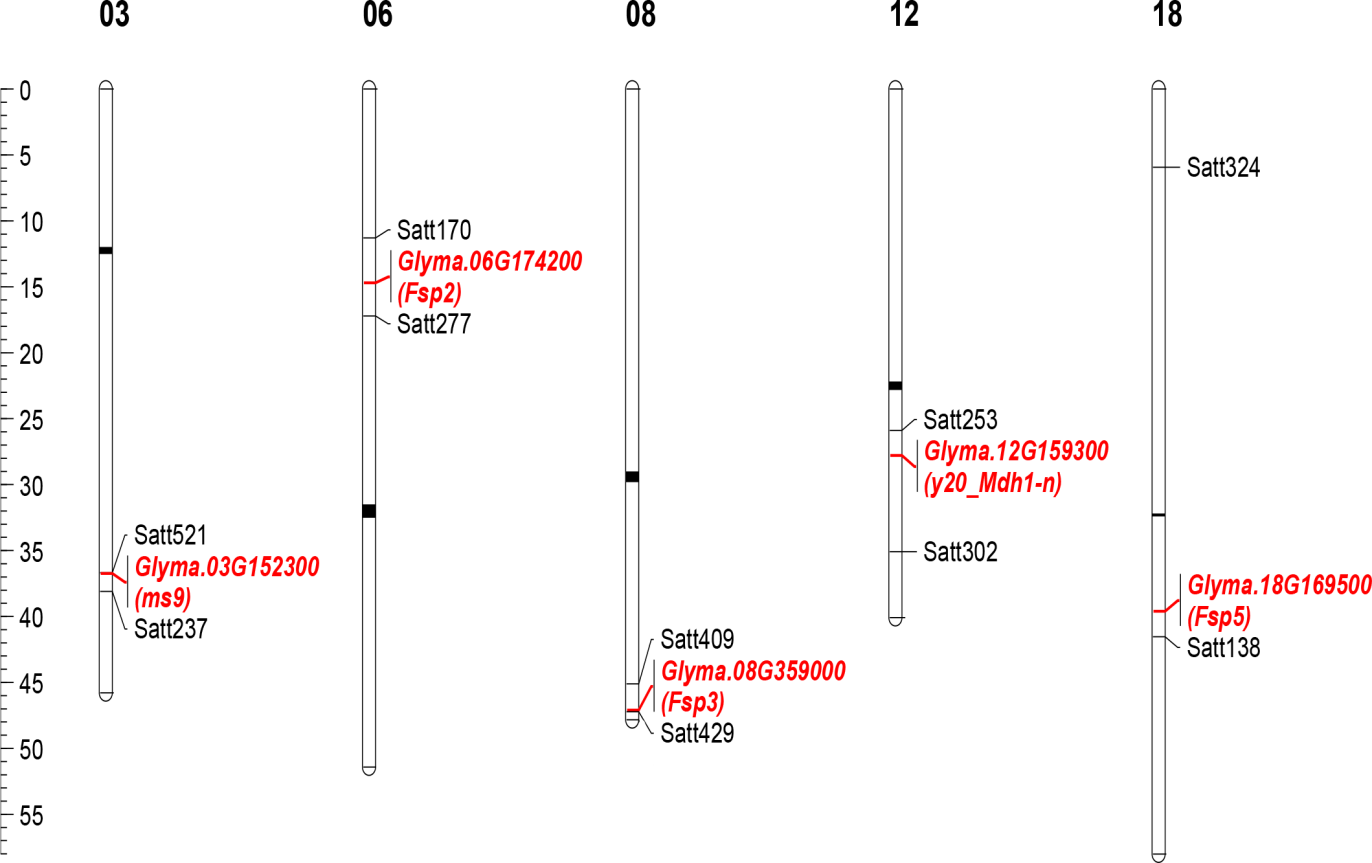
Physical map of five candidate genes that were mutated in *Tgm9*-insertion mutants. Flanking molecular markers from the genetic linkage maps for *y20 Mdh1-n*, *ms9*, *Fsp2*, *Fsp3* and *Fsp5* were placed on the physical map along with *Tgm9*-insertion sites identified in these mutants [29–31]. Scale is represented in million base pairs (Mb) of DNA.

The *Tgm9*-insertion sites were mapped to single genes in each of the five mutants investigated (Tables 1 and 2; Fig 1). The five *Tgm9*-induced mutant genes identified in these five mutants are from previously mapped mutant loci or regions. For example, in three chlorophyll deficient mutants (*y20 mdh1-n*) a *Tgm9* insertion was identified in the first intron of *Glyma*.*12G159300* encoding lactate/malate dehydrogenase (Table 2). The *y20 Mdh1-n* locus was originally mapped to Chromosome 12 and shown to be flanked by microsatellite markers Satt253 and Satt302 [29] (Table 1). *Glyma*.*12G159300* is located in this *y20 Mdh1-n* genomic region. Similarly, the *Tgm9*-insertion site in the *ms9* mutant was located in the first intron of *Glyma*.*03G152300* that has no functional annotation (Table 2). The gene is in the *ms9* region flanked by Satt521 and Satt237 (https://soybase.org) [30, 32] (Tables 1 and 2; Fig 1). The *Tgm9*-insertion sites in *Fsp2* and *Fsp3* were detected in the 7^th^ intron and 2^nd^ exon of *Glyma*.*06G174200* and *Glyma*.*08G359000*, respectively (Table 2). *Glyma*.*06G174200* encodes a haloacid dehalogenase-like hydrolase; *Glyma*.*08G359000* encodes the embryo-specific protein 3, (ATS3) (Table 2) (https://soybase.org). The *Fsp2* locus is flanked by Satt170 and Satt277 on Chromosome 6 [31]. *Glyma*.*06G174200* is located in this *Fsp2* region (Tables 1 and 2; Fig 1). Likewise, the *Tgm9* insertion sites in the *fsp3* and *fsp5* mutants were also localized to the genomic regions to which *Fsp3* and *Fsp5* were genetically mapped (Fig 1; Table 2).

**Table 2:** Forward genetics approach showing identification of the *Tgm9* insertion sites in known soybean mutants identified from the progenies of mutable line T322 (*w4-m*).

Name of line	Mutant	Insertion site	Gene mutated	Upstream/ Exon/ Intron/ Downstream	Chromosome location of the gene	Annotation	Confirmation of the location
T323	*y20 (Ames 2) mdh1-n (Ames 2)*	Gm12: 27,790,785–27,790,786	*Glyma*.*12G159300*	1st Intron	Gm12: 27,787,400–27,791,365	Lactate/malate dehydrogenase	Located 1.9 Mb from Satt253
T325	*y20 (Ames 4)* *mdh 1-n (Ames 4)*	Gm12: 27,790,785–27,790,786	*Glyma*.*12G159300*	1st Intron	Gm12: 27,787,400–27,791,365	Lactate/malate dehydrogenase	Located 1.9 Mb from Satt253
T346	*y20 (Ames 17) mdh1-n (Ames 19)*	Gm12: 27,790,785–27,790,786	*Glyma*.*12G159300*	1st Intron	Gm12: 27,787,400–27,791,365	Lactate/malate dehydrogenase	Located 1.9 Mb from Satt253
T359	*ms9*	Gm03: 36,726,043–36,726,044	*Glyma*.*03G152300*	1st intron	Gm03: 367,24,492–36,727,791	No Functional annotation	Located 1.4 Mb from Satt237
T364	*fsp2*	Gm06: 14,708,172–14,708,173	*Glyma*.*06G174200*	7th intron	Gm06: 14,707,013–14,712,446	Haloacid dehalogenase-like hydrolase	Located 3.4 Mb from Satt170
T365	*fsp3*	Gm08: 47,100,615–47,100,616	*Glyma*.*08G359000*	2nd exon	Gm08: 47,099,991–47,102,594	Embryo-specific protein 3, (ATS3)	Located 115 kb from Satt429
T367	*fsp5*	Gm18: 39,611,234–39,611,235	*Glyma*.*18G169500*	272 bp upstream	Gm18: 39,611,506–39,611,853	Embryo-specific protein 3, (ATS3)	Located between satt324 and satt138

### 3.2. *Tgm9* transposes to all 20 soybean chromosomes

To better understand the transposition patterns of *Tgm9*, we identified 124 germinal revertants bearing only purple flowers, each from individual T322 (*w4-m*) mutable plants (S1 Fig). Therefore, each revertant was unique. We were able to determine insertion sites from 105 mutants (S3 Table). Whether *Tgm9* failed to insert into new genetic loci or we failed to recover the insertion sites among the remaining 19 mutants is unknown.

**Fig 2:**
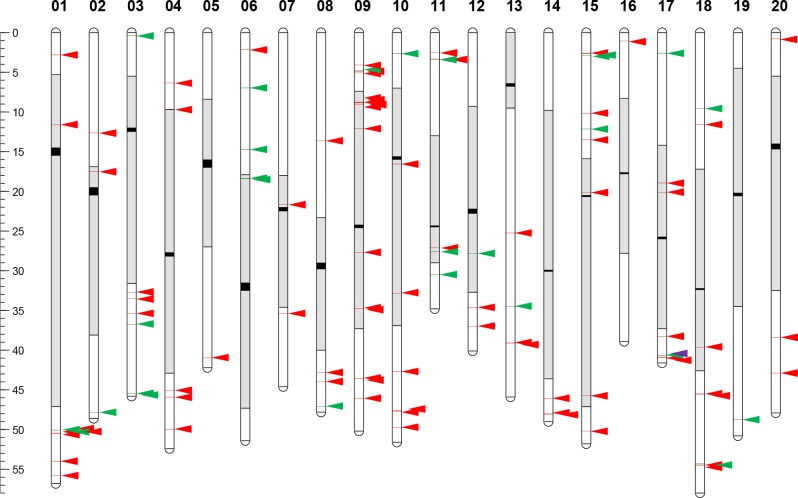
Physical map of the *Tgm9* insertion sites among 105 mutants. Green arrows represent locations of *Tgm9* in genes (exons and introns) and red arrows show *Tgm9* insertions in other genomic regions. A purple arrow shows the location of *Tgm9* in *W4* on Chromosome 17. Centromeres (black rectangles) and heterochromatic regions (grey areas) are shown on individual chromosomes. Scale is represented in million base pairs (Mb) of DNA.

Physical mapping of the *Tgm9* insertion sites among 105 mutants revealed that the element transposes to all 20 chromosomes (Fig 2). Insertion sites per chromosome ranged from 1 to 16 per chromosome (Fig 2). These vast differences in the distribution of insertions among chromosomes could be due to the small sample size of the population studied or due to a preference of the element to transpose into some genomic regions. There were six genomic regions, <1 Mb in size, which were enriched in *Tgm9* insertions. Of the 105 identified mutants, 24 were mapped to these six regions: (i) Chromosome 1 (six *Tgm9* insertions between 50.06 and 50.50 Mb region (http://www.soybase.org/SequenceIntro.php); (ii) Chromosome 9 (four between 4.07 and 5.07 Mb); (iii) Chromosome 9 (five between 8.77 and 9.06 Mb), (iv) Chromosome 15 (three between 2.58 and 2.87 Mb); (v) Chromosome 17 (three between 40.65 and 40.89 Mb); and (vi) Chromosome 18 (three between 54.31 and 54.51 Mb) (Fig 2). In soybean the euchromatic region constitutes 43% of the genome [1]. In this study, *Tgm9* transposed to euchromatic regions 77.1% of the time. The remaining 22.9% of insertions were mapped to the pericentromeric regions (Fig 2).

### 3.3. Organization of *Tgm9-*induced mutations in the soybean genome

**Fig 3:**
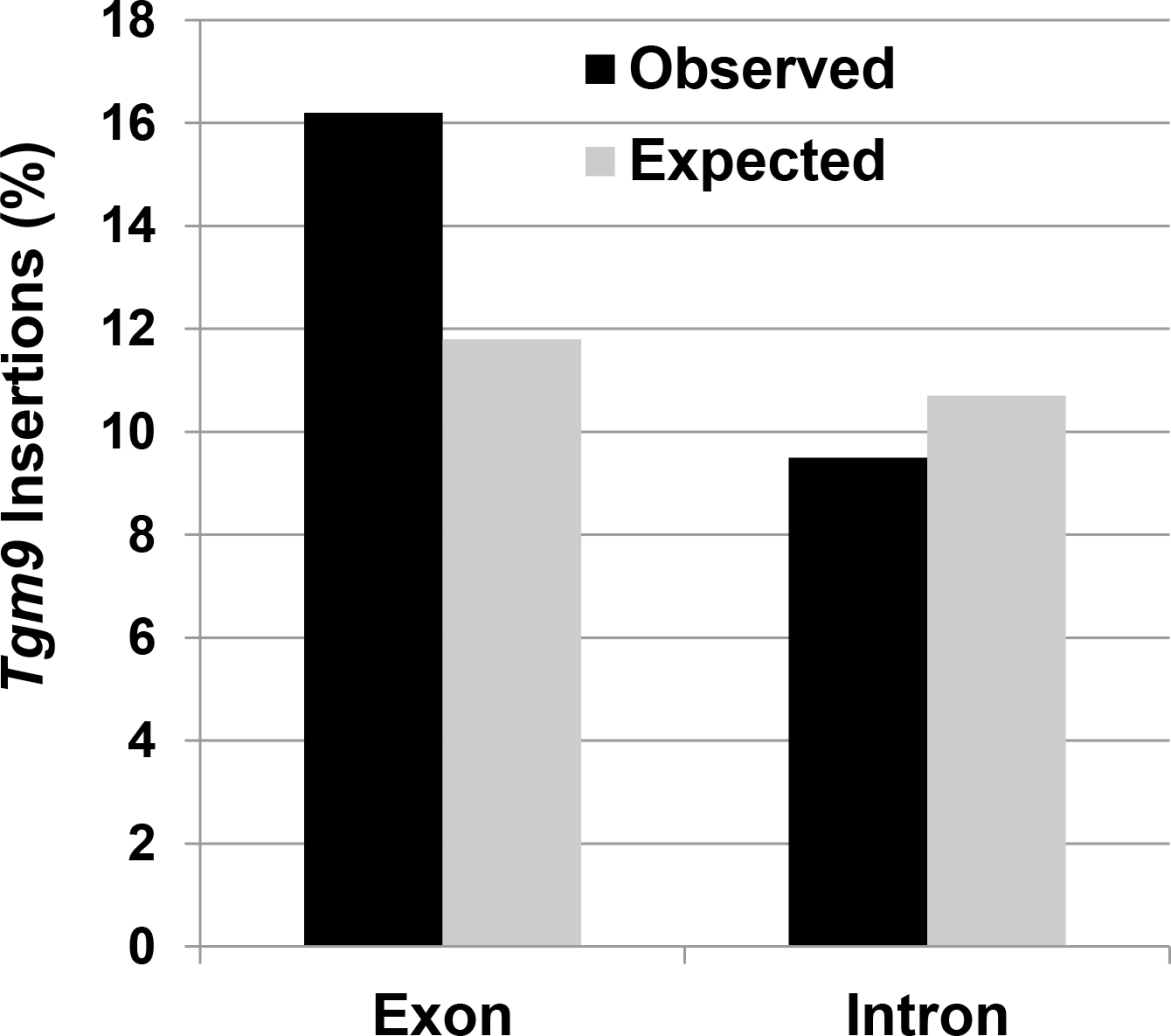
Percent distribution of the type of *Tgm9*-induced mutants. The observed frequency of *Tgm9* insertions in exonic and intronic sequences were calculated from 105 *Tgm9* insertion loci (S3 Table). The expected frequency of *Tgm9* is based on random insertion of the element in the exonic and intronic sequences calculated from the soybean genome sequences mapped to chromosomes (Soybase). The χ^2^ value (1.7752 at df = 1) calculated for observed *Tgm9* insertion frequencies and expected frequencies of random *Tgm9* insertions in exon and intron sequences is statistically non-significant at *p* = 0.05.

To study the distribution of transposition events of *Tgm9* across the chromosomes, the physical locations of the *Tgm9* insertion sites in the soybean genome were investigated. Although *Tgm9*-insertion in promoters can alter phenotypes, due to the limitation in precisely predicting the promoter sequences we calculated the number of insertions that were localized to only exon- and intron-sequences. These mutations are expected to knock-out gene function, and therefore are desired mutations for functional analyses of soybean genes. Of the 105 insertions studied, 16.2% and 9.5% of the *Tgm9*-insertions were localized to exons and introns, respectively (Fig 3, S3 Table). Thus, 25.7% of the insertions were generated in gene-sequences. In the soybean genome, gene-sequences cover 22.1% of the genome (S3 Table), which is very comparable to the observed insertion rate of 25.7% in the gene-sequences. The calculated χ^2^ value (1.92 at df = 1) suggests that the observed frequencies of *Tgm9* insertions in exons and introns (Fig 3) is statistically not significant at *p* = 0.05 from the expected values resulting from random insertion of the element in exon and intron sequences. We therefore conclude based on the study of 105 mutants that the *Tgm9* element appears to transpose randomly in the soybean genome (Fig 3, S3 Table).

## 4. Discussion

The availability of the genome sequence has greatly expedited molecular and genomic research in soybean [1]. Reverse genetic approaches such as TILLING and deletion mutants induced by fast neutron irradiation are being exploited for functional characterization of the soybean genome [33, 34]. Heterologous transposable elements have also been successfully applied in functional characterization of soybean genes [11–13].

The *Ac/Ds* transposon system in maize causes insertion mutations in closely linked locations. It has been applied to target specific chromosomal regions in several plant species [35, 36]. In soybean, the feasibility of *Ac/Ds* was tested by sequencing flanking regions of 200 individual mutants and was validated by isolating a gene involved in male-fertility [13]. Similarly, *mPing*, a miniature inverted repeat transposable element (MITE) from rice was evaluated in soybean [12]. *mPing* was shown to transpose to unlinked regions with a strong preference for gene-containing regions [12]. *Tnt1*, a tobacco retrotransposon, was effectively transformed into soybean and was shown to be re-activated by tissue culture [11].

Transposon-induced mutants are preferred over mutants generated using irradiation or chemical mutagenesis approaches because they typically contain a very few mutations per mutant and therefore are easier to analyze and utilize in breeding programs. However, heterologous transposable elements have to be genetically transformed and at least for the maize *Ac/Ds* system requires the generation of a large collection of transgenic soybean lines because the element moves only to linked genomic regions [37]. The requirement of tissue culture for the activation of *Tnt1* limits its uses in functional analyses of soybean genes because tissue culture itself can generate new genetic and epigenetic mutations, broadly known as somaclonal variation. Furthermore, functional analyses of tens of thousands of soybean genes will require growing a huge number of transgenic soybean lines in the field with proper care not to release transgenes to the environment. Once a product is developed from such a huge effort, the seed industry must then wait to get approval for deregulating the transgenic soybean lines. Then, there is also the issue of non-acceptance of transgenic soybeans by a significant proportion of soybean consumers. Given the complexities associated with the use of heterologous transposons summarized above, endogenous transposable elements are better suited for functional analyses of soybean genes and the generation of useful mutants for breeding desirable cultivars.

Several endogenous transposable elements such as *Tgm1*, *Tgm2*, *Tgm3*, *Tgm4*, *Tgm5*, *Tgm6*, *Tgm7*, *Tgm8*, *Tgm9*, Tgmt* and TgmR* have been identified in soybean [14, 38–40]. Of these, *Tgm9* has been shown to be active and has recently been used to clone a male-sterile, female-sterile gene (*GmMER3*) that encodes an ATP dependent DNA helicase [14, 41, 42]. It is possible, although unlikely, to have another *Tgm* sequence(s) causing mutation in *GmMER3* or other genes investigated in this study. To determine if the mutation in the male-sterile, female-sterile (*mer3*) mutant was caused by *Tgm9*, and not by any other active elements such as Tgmt*, the 3’-end 2,523 bp region of the transposon in the *GmMER3* gene of the *mer3* mutant was amplified and sequenced. The sequence matched perfectly with the *Tgm9* sequence suggesting that the *mer3* mutation was caused by *Tgm9* insertion, not by Tgmt* (S4 Fig). Furthermore, sequencing of the T322 genome revealed that *Tgm9* is the only intact CACTA-type element identified in this cultivar. In addition to the expected location of *Tgm9* in *W4*, an additional copy of the element was identified in a locus on Chromosome 19. This *Tgm9* element was hemizygous in T322 and absent among 15 selected *Tgm9*-induced mutants. This suggests that most likely the *Tgm9* copy on Chromosome 19 was generated from a recent transposition event (J. Baumbach, S. Srivastava and M.K. Bhattacharya, unpublished).

Here we have demonstrated that following excision from *dfr2*, *Tgm9* induces mutations in unlinked soybean genes (Fig 1). We have also demonstrated from analysis of 105 independent *Tgm9*-induced mutants that the element transposes to unlinked loci on all 20 soybean chromosomes (Fig 2). Since *Tgm9* is the only intact CACTA-type element in T322, the mutant genes or sequences identified in this study were resulted from insertion of *Tgm9*. To our knowledge, *Tgm9* is only reported active transposable element in soybean [14].

**Fig 4:**
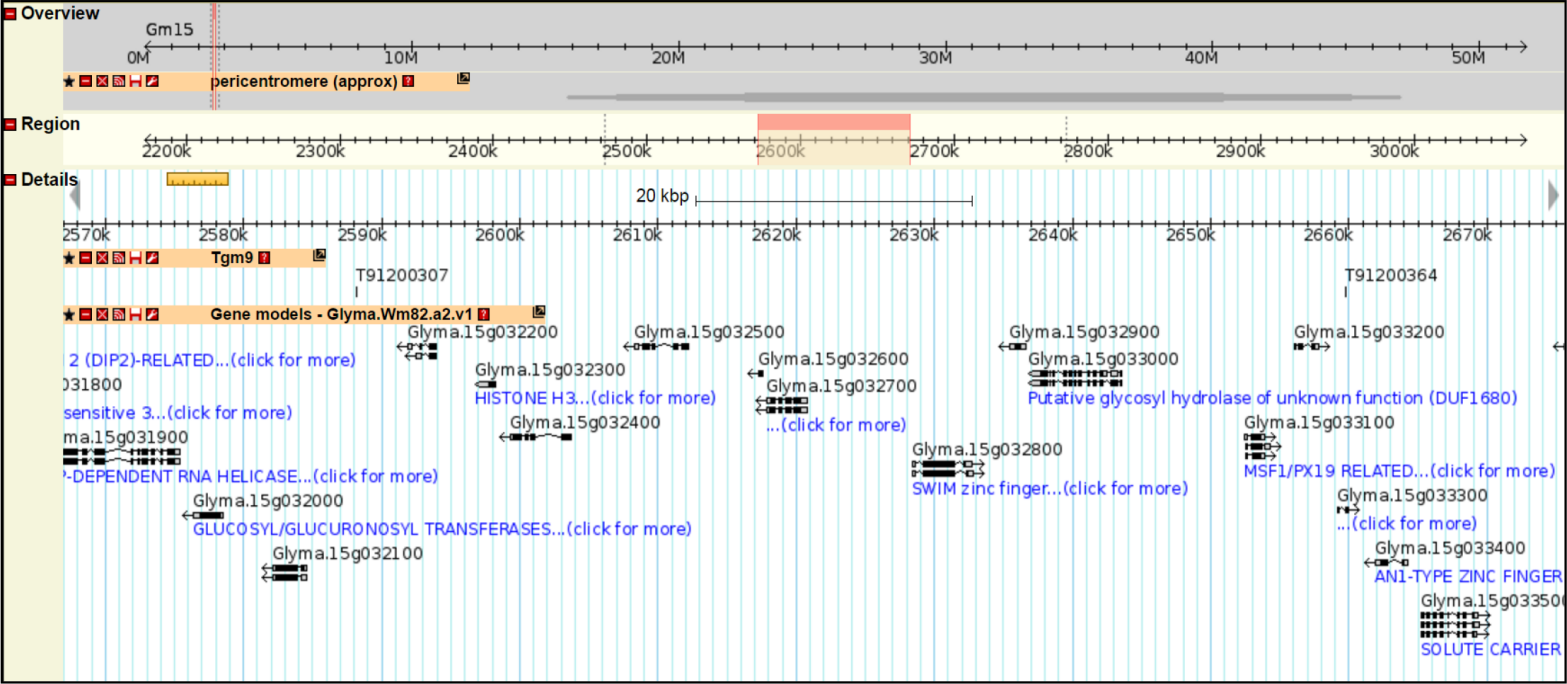
Display of *Tgm9*-induced mutants in the SoyBase genome browser. The track for *Tgm9*-insertion lines shows the location of two *Tgm9*-induced insertions (T91200307 and T91200364) on Chromosome 15. Note that in the T91200307 mutant, the *Tgm9* insertion site is located in the inter-genic region, and in T91200364, the insertion site is in the 2^nd^ exon of *Glyma*.*15G033300*. (http://soybase.org/gb2/gbrowse/gmax2.0x/?start=1;stop=56831624;ref=Gm01;width=1024;version=100;flip=0;grid=1;id=28f86f22137273c155fe3e1756259be0;l=tgm9-gene_models_wm82_a2_v1-pericentromere%3Aoverview {Please use user name: Bhatt; password: tgm9#}).

In this study we have observed that *Tgm9* transposes into unlinked loci following excision from the *w4-m* mutant allele and that such transpositions could cause functional mutations in over 25% of the mutants. There are 15,166 single copy genes in the soybean genome and *Tgm9* should be a suitable tool for generating mutations in most of these genes [1]. However, *Tgm9* has preference for certain genomic regions (Fig 2). This will reduce the efficiency of *Tgm9* in inducing mutations in such a study for functional analyses of soybean genes. Utilizing *Tgm9* to advance soybean genetics and biology will therefore require a large indexed *Tgm9*-induced mutant population. These mutants can be easily identified by taking the advantage of next generation sequencing platforms [7, 43] and displayed in the SoyBase genome browser (Fig 4). The ability of *Tgm9* to precisely excise from mutant loci for reconstituting the wild-type function may eliminate the need of complementation analyses of mutants through genetic transformation for identification of novel genes [42].

The 20,548-bp *Tgm9* element has been reported to be fractured during transposition, leading to the generation of stable mutants which could be used in soybean breeding programs [14]. Furthermore, cultivars generated by using such *Tgm9-*induced mutants can reach consumers rapidly since they do not require the lengthy deregulation process of mutants generated from the use of heterologous transposon systems.

## 5. Conclusions

In this investigation we have shown that *Tgm9* is an active transposable element that can induce mutations in unlinked genes on all 20 soybean chromosomes. It appears based on the study of 105 mutants that the *Tgm9* element randomly transposes into genes and over 25% of the mutants are expected to be knockout mutants, suitable for functional analyses of soybean genes. As there is a single active copy of this element in the mutable T322 line, identification of the insertion sites in a large collection of *Tgm9*-induced mutants is feasible through application of a next-generation sequencing platform. In soybean, the functions of the majority of the genes are still unknown. The generation of an indexed transposon-induced mutant population using *Tgm9* and their display in the SoyBase genome browser in the context of the other information available at SoyBase (Fig 4) will likely facilitate the functional characterization of most of the 15,166 single copy soybean genes [1]. Desirable *Tgm9*-induced mutant genes carrying inactive, fractured *Tgm9*-elements can also be identified and incorporated into elite breeding programs leading to relatively rapid release of genetically improved cultivars for commercial cultivation.

## Supporting information

S1 Fig Identification of a germinal revertant from a progeny row of a single mutable T322 (*w4-m*) plant. Approximately 150 progeny of a mutable plant identified in an earlier experiment were grown in a 15-foot long plot to locate a germinal revertant with only purple flowers.
(PPTX)

S2 Fig Flow diagram of the transposon display procedure. Adaptor has 5’ extended strand with no binding site for primers AP1 (Adaptor Primer 1) or AP2 (Adaptor Primer 2). Binding site for AP1 or AP2 can only be generated by transposon specific primers (TransR1 or TransR2). Exposed 3’ end of the adaptor is blocked by amino group to prevent extension. Unique bands from different lanes are excised and sequenced. The bands that are common to all lanes are most likely ancient transposition events.
(PPTX)

S3 Fig Transposon display of two selected progenies from each of six independent plants carrying variegated flowers. A single progeny row was grown from each of the six independent mutable plants harvested in 2014. From each row, two plants were selected for transposon display: (i) one plant with only green stem; (ii) the other plant with only purple stem (germinal revertant). The PCR fingerprints of each of the six plants with only green stem are shown on lanes 1 through 6; and those for six plants with purple stem on lanes 7 through 12. Note that two plants, Plant # 1 under green stem heading and Plant # 1 under purple stem heading, were descended from the same mutable plant harvested in 2014. Two restriction endonucleases, *Eco*RV and *Pvu*II, were used in digesting the genomic DNA for generating the transposon displays. White arrows show the amplification of some of the residual insertions; whereas, red arrows indicate the progeny-specific amplification presumably from new *Tgm9* insertion sites in distinct loci. *DFR2*-specific amplification was observed for the plants with purple stems (germinal revertants). Note that in Plant # 5 with purple stem failed to amplify the *Tgm9* insertion site at the *DFR2* intron II presumably because of simultaneous *Tgm9* excision from both *DFR2* copies. The sibling plants with green stems failed to amplify *DFR2* because of the presence of *Tgm9* in both *DFR2* copies. Sub-PCR of the two strong PCR amplified ~750 bp fragments in Plant # 3 and 6 with green stem using *DFR2* F and TransR2 primers (Supplemental Table 1) indicated that the intense amplified PCR products were from the *DFR2* locus (data are not shown).
(PPTX)

S4 Fig *Tgm9* caused the insertion mutation in the *GmMER3* gene of the *mer3* mutant. Transposon insertion sequence from the *GmMER3* gene was amplified by conducting long-range PCR and compared to both *Tgm9* and Tgmt*, the two highly similar transposons characterized from soybean. A) The orientation of the *Tgm9* insertion in the *MER3* gene and primers used for nested PCR and sequencing are shown. B) First PCR product was amplified using primers 1 and 2. The amplified PCR product was 2,823 bp. C) The nested PCR product was amplified using primers 3 and 4. The amplified PCR product was 2,758 bp. Primer 3 was used for sequencing the PCR product represented by the dashed line X. Primer 4 was used to sequence the PCR product and produced sequence represented by the dashed line Y. D) Sequence from primer 3 matched the *MER3* gene and the start of the 5’ end of the *Tgm9* transposon sequence shown with the red font. E) Sequence from primer 4 aligned to the *Tgm9* and Tgmt*. The PCR product matches the *Tgm9* sequence perfectly. Polymorphic nucleotides between the insertion sequence and Tgmt* sequence are shown in red font.
(PPTX)

S1 Table List of primers used in this investigation. (DOCX)

S2 Table Exon and intron sequences of the soybean genome. (DOCX)

S3 Table *Tgm9* insertion sites in 105 independent mutants. (XLSX)

## Abbreviations

DFR: Dihydroflavonol-4-reductase
RNAi: RNA interference
TILLING: Targeting Induced Local Lesion in Genomics
MITE: Miniature Inverted Repeat Transposable Element
