# Supplementary material for: The endogenous transposable element Tgm9 is suitable for generating knockout mutants for functional analyses of soybean genes and genetic improvement in soybean
Source: PLoS One. 2017 Aug 10;12(8):e0180732. doi: 10.1371/journal.pone.0180732 (PMC5552171; doi:10.1371/journal.pone.0180732)
Supplement: S1 Fig — Approximately 150 progeny of a mutable plant identified in an earlier experiment were grown in a 15-foot long plot to locate a germinal revertant with only purple flowers. (PPTX) [file pone.0180732.s001.pptx]

## Slide 1
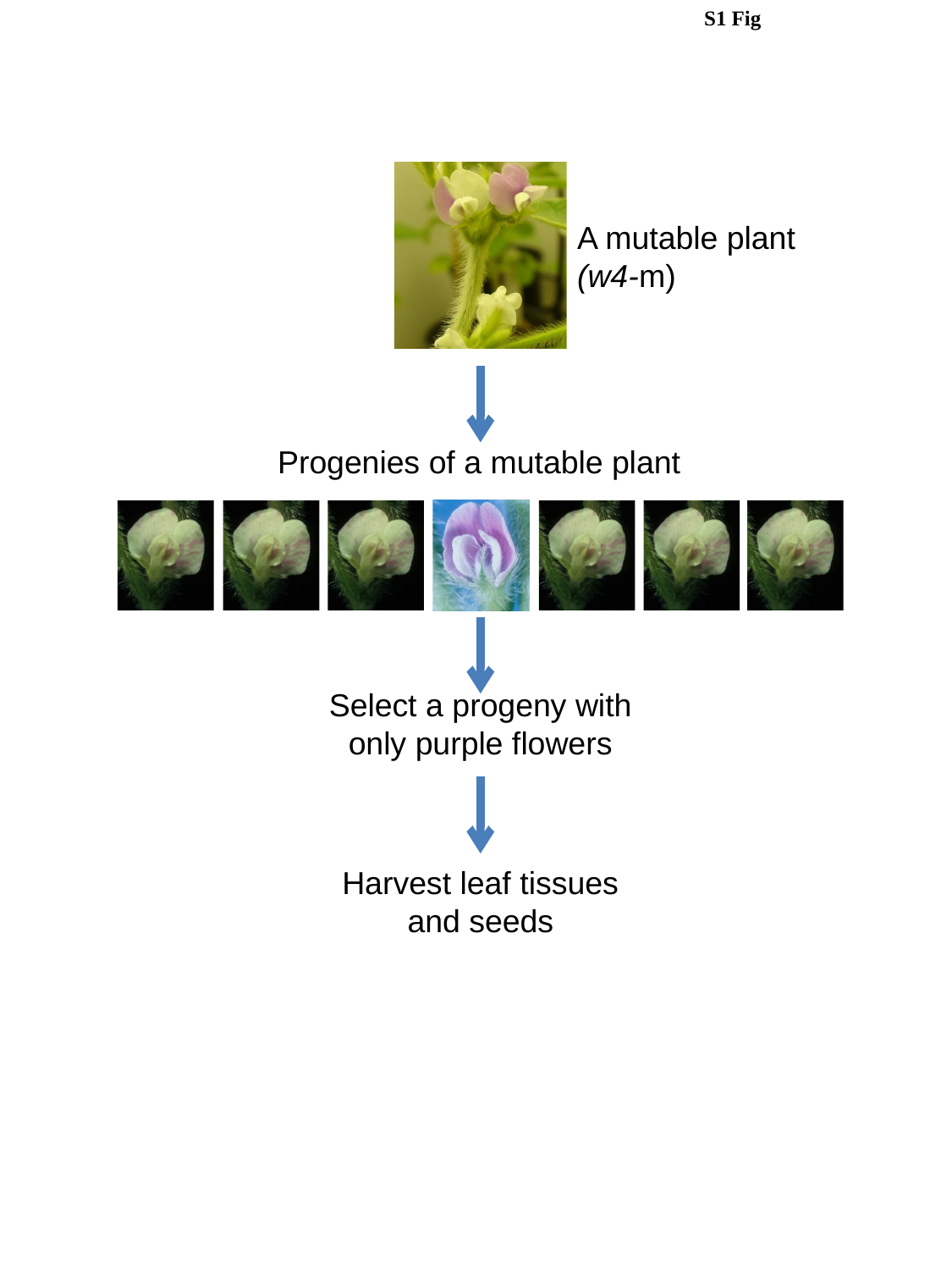

S1 Fig
A mutable plant (w4-m)
Progenies of a mutable plant
Select a progeny with only purple flowers
Harvest leaf tissues and seeds
