# Supplementary material for: The endogenous transposable element Tgm9 is suitable for generating knockout mutants for functional analyses of soybean genes and genetic improvement in soybean
Source: PLoS One. 2017 Aug 10;12(8):e0180732. doi: 10.1371/journal.pone.0180732 (PMC5552171; doi:10.1371/journal.pone.0180732)
Supplement: S2 Fig — Adaptor has 5’ extended strand with no binding site for primers AP1 (Adaptor Primer 1) or AP2 (Adaptor Primer 2). Binding site for AP1 or AP2 can only be generated by transposon specific primers (TransR1 or TransR2). Exposed 3’ end of the adaptor is blocked by amino group to prevent extension. Unique bands from different lanes are excised and sequenced. The bands that are common to all lanes are most likely ancient transposition events. (PPTX) [file pone.0180732.s002.pptx]

## Slide 1
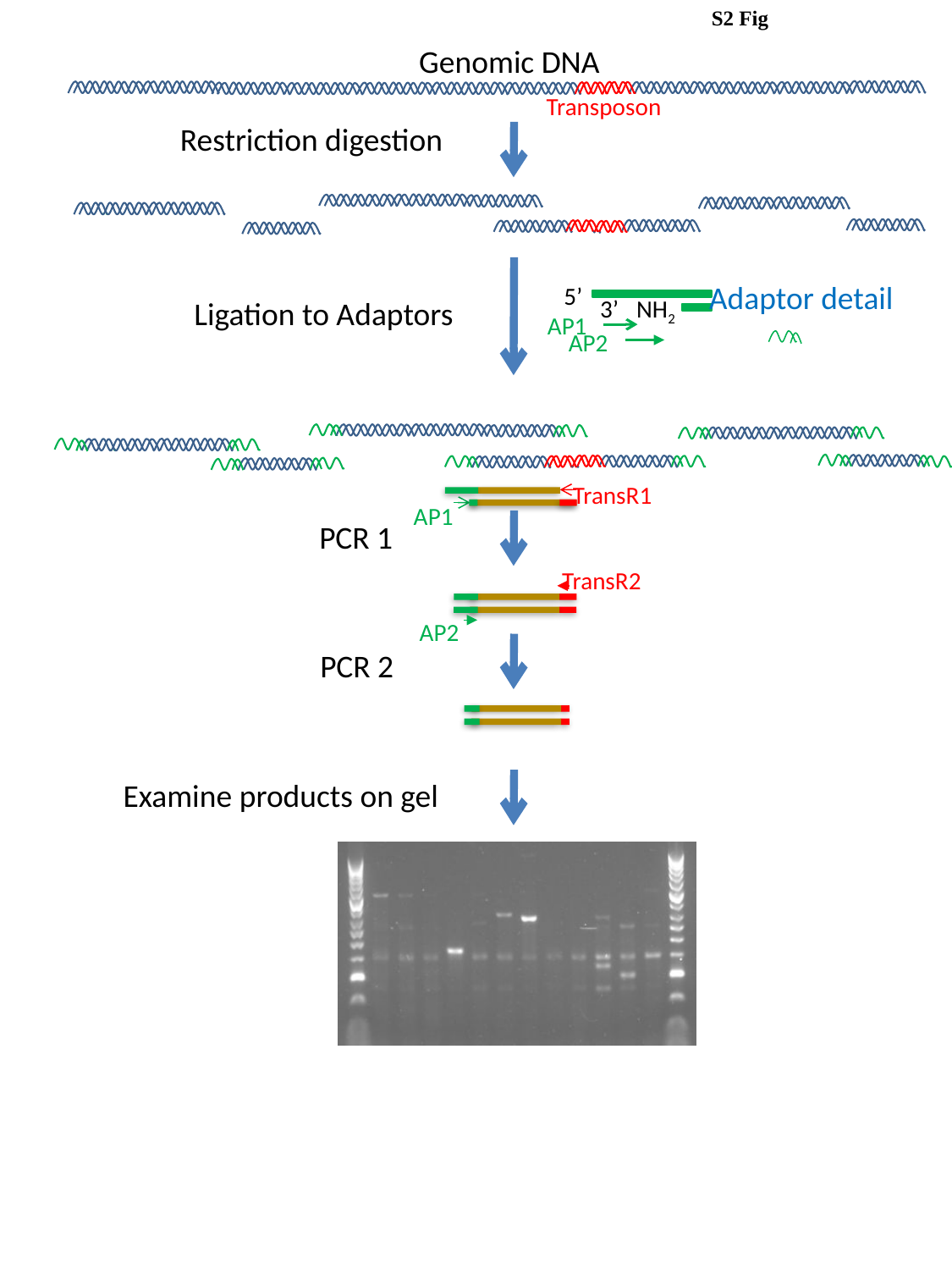

S2 Fig
Genomic DNA
Transposon
Restriction digestion
Adaptor detail
5’
NH2
3’
Ligation to Adaptors
AP1
AP2
TransR1
AP1
PCR 1
TransR2
AP2
PCR 2
Examine products on gel
