# Supplementary figures and images for: The endogenous transposable element Tgm9 is suitable for generating knockout mutants for functional analyses of soybean genes and genetic improvement in soybean
Source: PLoS One. 2017 Aug 10;12(8):e0180732. doi: 10.1371/journal.pone.0180732 (PMC5552171; doi:10.1371/journal.pone.0180732)

## Slide 1
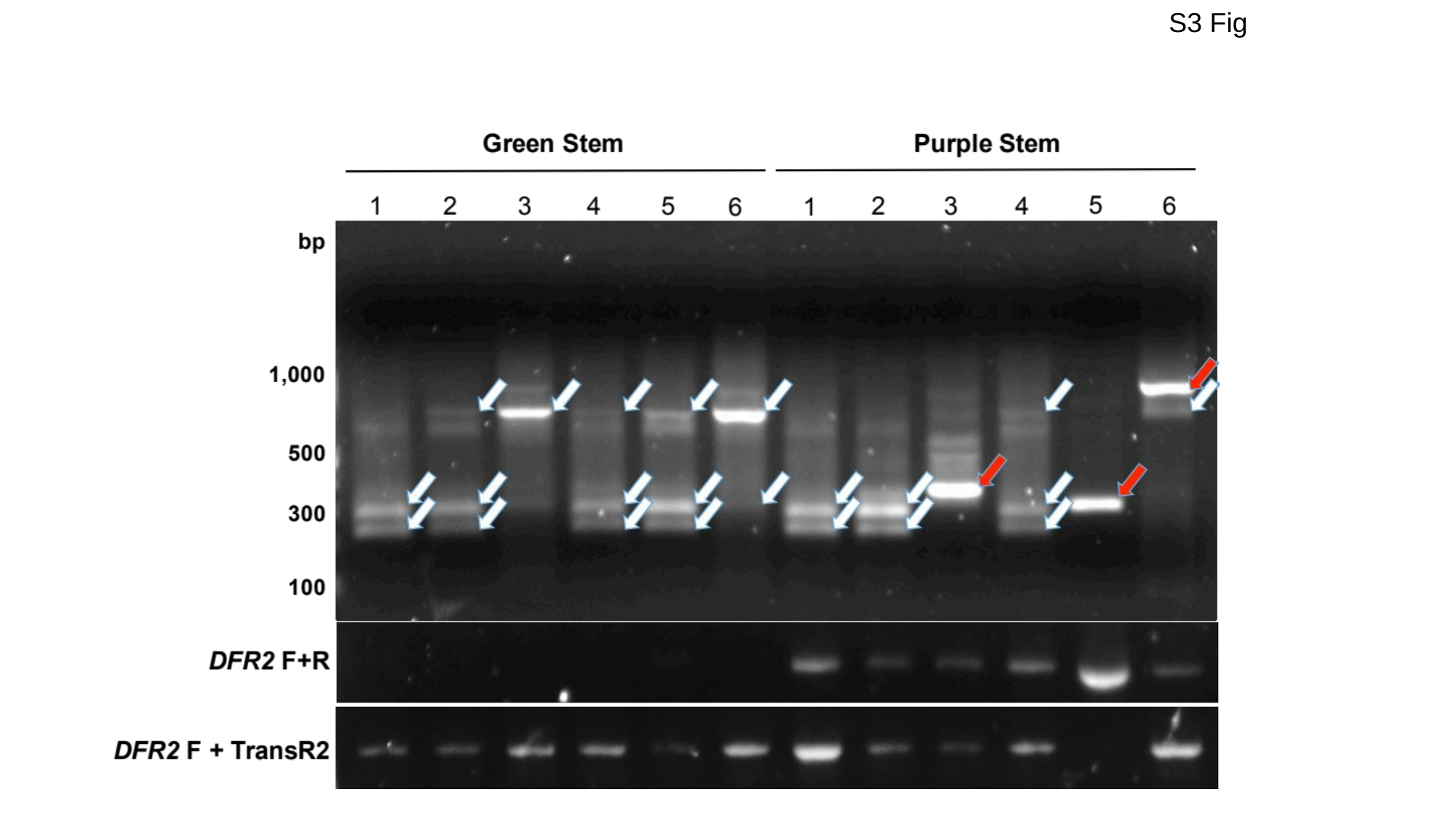

S3 Fig

Supplement: S3 Fig — A single progeny row was grown from each of the six independent mutable plants harvested in 2014. From each row, two plants were selected for transposon display: (i) one plant with only green stem; (ii) the other plant with only purple stem (germinal revertant). The PCR fingerprints of each of the six plants with only green stem are shown on lanes 1 through 6; and those for six plants with purple stem on lanes 7 through 12. Note that two plants, Plant # 1 under green stem heading and Plant # 1 under purple stem heading, were descended from the same mutable plant harvested in 2014. Two restriction endonucleases, EcoRV and PvuII, were used in digesting the genomic DNA for generating the transposon displays. White arrows show the amplification of some of the residual insertions; whereas, red arrows indicate the progeny-specific amplification presumably from new Tgm9 insertion sites in distinct loci. DFR2-specific amplification was observed for the plants with purple stems (germinal revertants). Note that in Plant # 5 with purple stem failed to amplify the Tgm9 insertion site at the DFR2 intron II presumably because of simultaneous Tgm9 excision from both DFR2 copies. The sibling plants with green stems failed to amplify DFR2 because of the presence of Tgm9 in both DFR2 copies. Sub-PCR of the two strong PCR amplified ~750 bp fragments in Plant # 3 and 6 with green stem using DFR2 F and TransR2 primers (Supplemental Table 1) indicated that the intense amplified PCR products were from the DFR2 locus (data are not shown). (PPTX) [file pone.0180732.s003.pptx]
