# Supplementary material for: The endogenous transposable element Tgm9 is suitable for generating knockout mutants for functional analyses of soybean genes and genetic improvement in soybean
Source: PLoS One. 2017 Aug 10;12(8):e0180732. doi: 10.1371/journal.pone.0180732 (PMC5552171; doi:10.1371/journal.pone.0180732)
Supplement: S4 Fig — Transposon insertion sequence from the GmMER3 gene was amplified by conducting long-range PCR and compared to both Tgm9 and Tgmt*, the two highly similar transposons characterized from soybean. A) The orientation of the Tgm9 insertion in the MER3 gene and primers used for nested PCR and sequencing are shown. B) First PCR product was amplified using primers 1 and 2. The amplified PCR product was 2,823 bp. C) The nested PCR product was amplified using primers 3 and 4. The amplified PCR product was 2,758 bp. Primer 3 was used for sequencing the PCR product represented by the dashed line X. Primer 4 was used to sequence the PCR product and produced sequence represented by the dashed line Y. D) Sequence from primer 3 matched the MER3 gene and the start of the 5’ end of the Tgm9 transposon sequence shown with the red font. E) Sequence from primer 4 aligned to the Tgm9 and Tgmt*. The PCR product matches the Tgm9 sequence perfectly. Polymorphic nucleotides between the insertion sequence and Tgmt* sequence are shown in red font. (PPTX) [file pone.0180732.s004.pptx]

## Slide 1
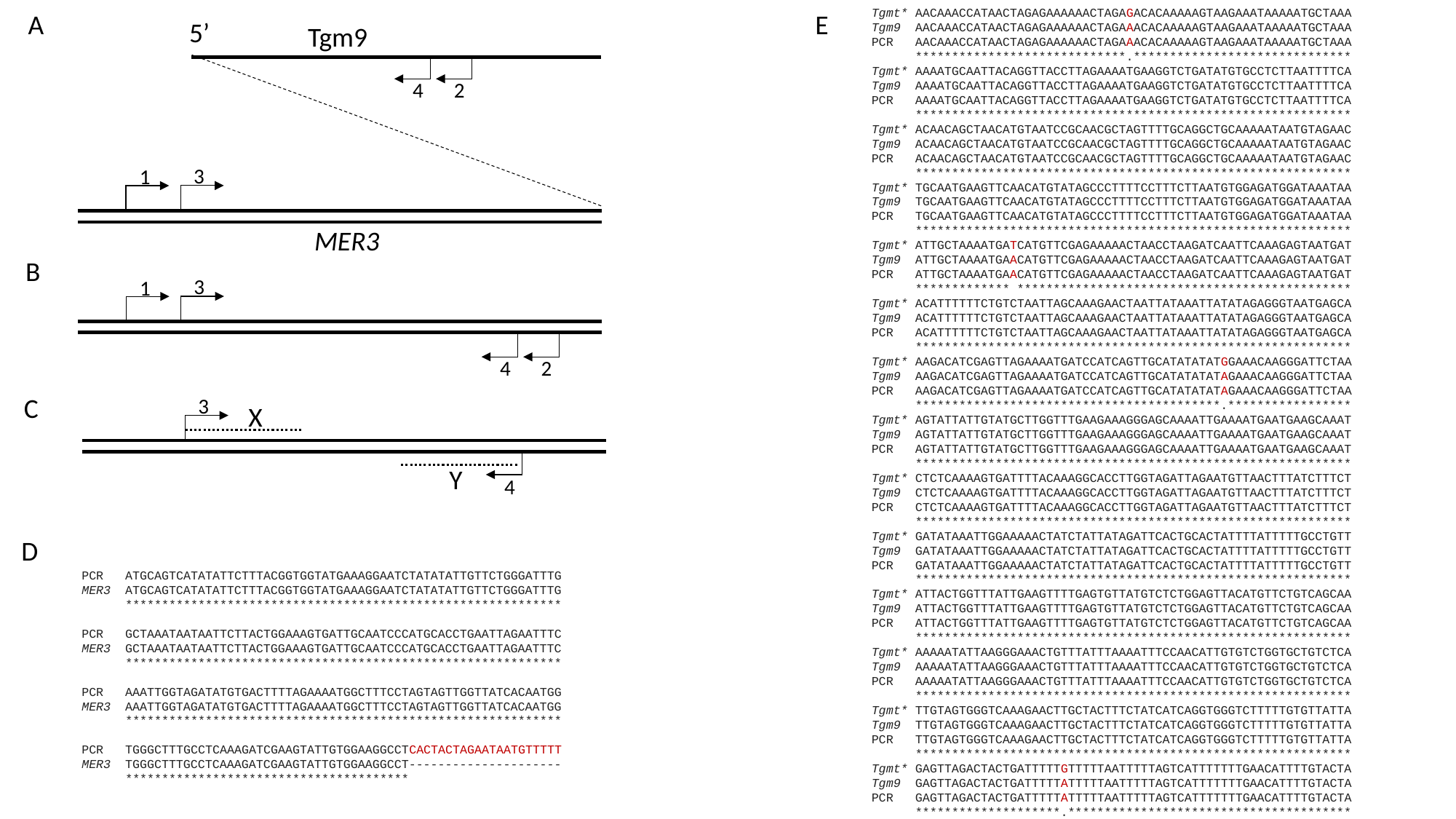

S4 Fig
Tgmt* AACAAACCATAACTAGAGAAAAAACTAGAGACACAAAAAGTAAGAAATAAAAATGCTAAA
Tgm9 AACAAACCATAACTAGAGAAAAAACTAGAAACACAAAAAGTAAGAAATAAAAATGCTAAA
PCR AACAAACCATAACTAGAGAAAAAACTAGAAACACAAAAAGTAAGAAATAAAAATGCTAAA
 *****************************.******************************
Tgmt* AAAATGCAATTACAGGTTACCTTAGAAAATGAAGGTCTGATATGTGCCTCTTAATTTTCA
Tgm9 AAAATGCAATTACAGGTTACCTTAGAAAATGAAGGTCTGATATGTGCCTCTTAATTTTCA
PCR AAAATGCAATTACAGGTTACCTTAGAAAATGAAGGTCTGATATGTGCCTCTTAATTTTCA
 ************************************************************
Tgmt* ACAACAGCTAACATGTAATCCGCAACGCTAGTTTTGCAGGCTGCAAAAATAATGTAGAAC
Tgm9 ACAACAGCTAACATGTAATCCGCAACGCTAGTTTTGCAGGCTGCAAAAATAATGTAGAAC
PCR ACAACAGCTAACATGTAATCCGCAACGCTAGTTTTGCAGGCTGCAAAAATAATGTAGAAC
 ************************************************************
Tgmt* TGCAATGAAGTTCAACATGTATAGCCCTTTTCCTTTCTTAATGTGGAGATGGATAAATAA
Tgm9 TGCAATGAAGTTCAACATGTATAGCCCTTTTCCTTTCTTAATGTGGAGATGGATAAATAA
PCR TGCAATGAAGTTCAACATGTATAGCCCTTTTCCTTTCTTAATGTGGAGATGGATAAATAA
 ************************************************************
Tgmt* ATTGCTAAAATGATCATGTTCGAGAAAAACTAACCTAAGATCAATTCAAAGAGTAATGAT
Tgm9 ATTGCTAAAATGAACATGTTCGAGAAAAACTAACCTAAGATCAATTCAAAGAGTAATGAT
PCR ATTGCTAAAATGAACATGTTCGAGAAAAACTAACCTAAGATCAATTCAAAGAGTAATGAT
 ************* **********************************************
Tgmt* ACATTTTTTCTGTCTAATTAGCAAAGAACTAATTATAAATTATATAGAGGGTAATGAGCA
Tgm9 ACATTTTTTCTGTCTAATTAGCAAAGAACTAATTATAAATTATATAGAGGGTAATGAGCA
PCR ACATTTTTTCTGTCTAATTAGCAAAGAACTAATTATAAATTATATAGAGGGTAATGAGCA
 ************************************************************
Tgmt* AAGACATCGAGTTAGAAAATGATCCATCAGTTGCATATATATGGAAACAAGGGATTCTAA
Tgm9 AAGACATCGAGTTAGAAAATGATCCATCAGTTGCATATATATAGAAACAAGGGATTCTAA
PCR AAGACATCGAGTTAGAAAATGATCCATCAGTTGCATATATATAGAAACAAGGGATTCTAA
 ******************************************.*****************
Tgmt* AGTATTATTGTATGCTTGGTTTGAAGAAAGGGAGCAAAATTGAAAATGAATGAAGCAAAT
Tgm9 AGTATTATTGTATGCTTGGTTTGAAGAAAGGGAGCAAAATTGAAAATGAATGAAGCAAAT
PCR AGTATTATTGTATGCTTGGTTTGAAGAAAGGGAGCAAAATTGAAAATGAATGAAGCAAAT
 ************************************************************
Tgmt* CTCTCAAAAGTGATTTTACAAAGGCACCTTGGTAGATTAGAATGTTAACTTTATCTTTCT
Tgm9 CTCTCAAAAGTGATTTTACAAAGGCACCTTGGTAGATTAGAATGTTAACTTTATCTTTCT
PCR CTCTCAAAAGTGATTTTACAAAGGCACCTTGGTAGATTAGAATGTTAACTTTATCTTTCT
 ************************************************************
Tgmt* GATATAAATTGGAAAAACTATCTATTATAGATTCACTGCACTATTTTATTTTTGCCTGTT
Tgm9 GATATAAATTGGAAAAACTATCTATTATAGATTCACTGCACTATTTTATTTTTGCCTGTT
PCR GATATAAATTGGAAAAACTATCTATTATAGATTCACTGCACTATTTTATTTTTGCCTGTT
 ************************************************************
Tgmt* ATTACTGGTTTATTGAAGTTTTGAGTGTTATGTCTCTGGAGTTACATGTTCTGTCAGCAA
Tgm9 ATTACTGGTTTATTGAAGTTTTGAGTGTTATGTCTCTGGAGTTACATGTTCTGTCAGCAA
PCR ATTACTGGTTTATTGAAGTTTTGAGTGTTATGTCTCTGGAGTTACATGTTCTGTCAGCAA
 ************************************************************
Tgmt* AAAAATATTAAGGGAAACTGTTTATTTAAAATTTCCAACATTGTGTCTGGTGCTGTCTCA
Tgm9 AAAAATATTAAGGGAAACTGTTTATTTAAAATTTCCAACATTGTGTCTGGTGCTGTCTCA
PCR AAAAATATTAAGGGAAACTGTTTATTTAAAATTTCCAACATTGTGTCTGGTGCTGTCTCA
 ************************************************************
Tgmt* TTGTAGTGGGTCAAAGAACTTGCTACTTTCTATCATCAGGTGGGTCTTTTTGTGTTATTA
Tgm9 TTGTAGTGGGTCAAAGAACTTGCTACTTTCTATCATCAGGTGGGTCTTTTTGTGTTATTA
PCR TTGTAGTGGGTCAAAGAACTTGCTACTTTCTATCATCAGGTGGGTCTTTTTGTGTTATTA
 ************************************************************
Tgmt* GAGTTAGACTACTGATTTTTGTTTTTAATTTTTAGTCATTTTTTTGAACATTTTGTACTA
Tgm9 GAGTTAGACTACTGATTTTTATTTTTAATTTTTAGTCATTTTTTTGAACATTTTGTACTA
PCR GAGTTAGACTACTGATTTTTATTTTTAATTTTTAGTCATTTTTTTGAACATTTTGTACTA
 ********************.***************************************
A
E
5’
Tgm9
2
4
3
1
MER3
B
3
1
2
4
C
3
X
Y
4
D
PCR ATGCAGTCATATATTCTTTACGGTGGTATGAAAGGAATCTATATATTGTTCTGGGATTTG
MER3 ATGCAGTCATATATTCTTTACGGTGGTATGAAAGGAATCTATATATTGTTCTGGGATTTG
 ************************************************************
PCR GCTAAATAATAATTCTTACTGGAAAGTGATTGCAATCCCATGCACCTGAATTAGAATTTC
MER3 GCTAAATAATAATTCTTACTGGAAAGTGATTGCAATCCCATGCACCTGAATTAGAATTTC
 ************************************************************
PCR AAATTGGTAGATATGTGACTTTTAGAAAATGGCTTTCCTAGTAGTTGGTTATCACAATGG
MER3 AAATTGGTAGATATGTGACTTTTAGAAAATGGCTTTCCTAGTAGTTGGTTATCACAATGG
 ************************************************************
PCR TGGGCTTTGCCTCAAAGATCGAAGTATTGTGGAAGGCCTCACTACTAGAATAATGTTTTT
MER3 TGGGCTTTGCCTCAAAGATCGAAGTATTGTGGAAGGCCT---------------------
 ***************************************
