# Supplementary material for: The endogenous transposable element Tgm9 is suitable for generating knockout mutants for functional analyses of soybean genes and genetic improvement in soybean
Source: PLoS One. 2017 Aug 10;12(8):e0180732. doi: 10.1371/journal.pone.0180732 (PMC5552171; doi:10.1371/journal.pone.0180732)
Supplement: S1 Table — (DOCX) [file pone.0180732.s005.docx]

| Primer Name | Primer sequence |  |
| --- | --- | --- |
| AP1 | GTAATACGACTCACTATAGGGC |  |
| Trans R1 | CGTCGTGGGTGAAGAGTGGGTGAAGAGTG |  |
| AP2 | ACTATAGGGCACGCGTGGT |  |
| Trans R2 | GCCACCCAGCGAGTTACTAAGATG |  |
| Mer3 Outer | GGTCAATCAAATCCATTCATTAAGAAC |  |
| Tgm9 Outer | GCAACATCAACATCTAATATATCGATC |  |
| Mer3 Inner | GGCTGAGAGAGGCTTCTTTATCTTG |  |
| Tgm9 Inner | CGATCTCTCTCACACATAGTAAAATATC |  |

**S1 Table.** List of primers used in this investigation.
