# Supplementary material for: The endogenous transposable element Tgm9 is suitable for generating knockout mutants for functional analyses of soybean genes and genetic improvement in soybean
Source: PLoS One. 2017 Aug 10;12(8):e0180732. doi: 10.1371/journal.pone.0180732 (PMC5552171; doi:10.1371/journal.pone.0180732)
Supplement: S2 Table — (DOCX) [file pone.0180732.s006.docx]

|  | **Total bases in Wm82.a2 genome for each gene sub-part** | | | | | |  |
| --- | --- | --- | --- | --- | --- | --- | --- |
|  | **Genes** | **5' UTR** | **3'UTR** | **CDS** | **Exon** | **Intron** |  |
| All Wm82.a2 genome sequence | 215,991,420 | 13,240,615 | 20,503,428 | 79,479,855 | 113,223,898 | 102,767,522 |  |
| % of total genome sequence | 22.1 | 1.4 | 2.1 | 8.1 | 11.6 | 10.5 |  |
|  |  |  |  |  |  |  |  |
| Only Wm82.a2 chromosomes | 214,397,367 | 13,154,805 | 20,386,748 | 78,907,186 | 112,448,739 | 101,948,628 |  |
| % of total chromosome sequence | 22.6 | 1.4 | 2.1 | 8.3 | 11.8 | 10.7 |  |
|  |  |  |  |  |  |  |  |
| Total chromosome length | 949,183,385 |  |  |  |  |  |  |
| Total scaffold length | 29,311,887 |  |  |  |  |  |  |
| Chromosome + Scaffold length | 978,495,272 |  |  |  |  |  |  |
| Exon and intron proportions used in this study are highlighted with yellow. | | | |  |  |  |  |
| Entire W82.a2 genome sequence was used in finding location of *Tgm9* insertion sites and therefore highlighted values are used  in this study. | | | | | | | |

**S2 Table:** Exon and intron sequences of the soybean genome.
